# Context Dependent Benzodiazepine Modulation of GABA_A_ Receptor Opening Frequency

**DOI:** 10.2174/157015910790909467

**Published:** 2010-03

**Authors:** Matt T Bianchi

**Affiliations:** Neurology Department, Sleep Division, Massachusetts General Hospital, Wang 720, Boston, MA, 02114, USA

**Keywords:** Mechanism, ion channel, synaptic, extra-synaptic, affinity.

## Abstract

The anxiolytic, hypnotic, and anti-convulsant properties of benzodiazepines (BDZs) require modulation of distinct GABA_A_ receptor α-subtypes. BDZ modulation of GABA_A_ receptors is often described in terms of increased opening frequency, and contrasted with the increased open durations occurring with barbiturate modulation. Several studies spanning single channel, rapid kinetic, and whole cell techniques have suggested that BDZs effect this observed change in frequency through increased affinity for GABA. BDZ-sensitive αβγ isoforms exist at extrasynaptic as well as synaptic locations, where they encounter markedly different concentration and time-course of GABA exposure. Interestingly, this affinity-based mechanism (specifically, decreasing the GABA unbinding rate) is only predicted to increase opening frequency under conditions that allow the unbinding and rebinding cycles typical of prolonged exposure to low GABA concentrations, which are more likely to occur at extrasynaptic GABA_A_ receptors. In contrast, when rebinding is less likely, such as may occur in certain synaptic conditions, the number, but not the frequency, of channel openings increases in response to BDZ modulation. In conclusion, not only can multiple kinetic mechanisms alter channel opening frequency, but a single mechanism – increased affinity – impacts opening frequency differently under different contexts of GABA_A_ receptor activation.

## INTRODUCTION.

The therapeutic importance of benzodiazepines (BDZs) spans multiple clinical domains, including seizures, anxiety, insomnia, and muscle relaxation. After studies of recombinant receptors demonstrated that point mutations in α subtypes effectively compromise BDZ modulation (α1(H101R), α2(H101R), α3(H126R), and α5(H105R)), mice harboring these mutations (*via *knock-in) demonstrated that clinically relevant behavioral effects of BDZs were linked to GABA_A_ receptors containing these α subtypes. The α 1-containing receptors were linked to the sedative effects of BZDs [10, 47], and behavioral and EEG characteristics of sleep were insensitive to diazepam in these transgenic mice [52]. The new-generation BDZ site hypnotics (zolpidem, zaleplon, and eszopiclone) are relatively α1 selective, although pharmacokinetic benefits over classical BDZ ligands may also contribute to their improved therapeutic utility [12]. The α2 subtype has been associated with the anxiolytic effects of BZDs [29]. Despite provocative localization of α3 subtype in the thalamic reticular nucleus, the sedative and sleep-EEG effects of BDZs were intact in the α3 knock-in mice [24]. Tolerance to the sedating effects of BDZ (but not the sedation itself) was shown to depend on the α5 subtype [54]. Some overlap in functional roles among the α subtypes is likely, given findings such as the abolished sleep EEG effects of BDZs in the α2 knock-in [25], and the partial compromise of anticonvulsant activity of the α1 knock-in. Also, these knock-in mutations may affect baseline neurophysiology, as increased behavioral excitability in the α2 knock-in mice was reported [37]. Another possible concern involves the potential disruption of signaling by endogenous BDZs, although this topic remains controversial [9]. Although a human epilepsy mutation reported to abolish BDZ activity was supportive of an endozepine mechanism, BDZ sensitivity was subsequently confirmed [3]. 

## BDZ MODULATION OF GABA_A_ RECEPTOR AFFINITY

Early work at the whole cell  [7, 30] and single channel [46, 53] levels confirmed GABA_A_ receptors as the BDZ targets. Single GABA_A_ receptors from dorsal root ganglia demonstrated increased single-channel opening frequency in response to the BDZ diazepam, without any change in mean open duration. The increase in single channel frequency seen with BDZs is often contrasted with the effect of barbiturates: increased open duration without altering opening frequency [31, 50, 53]. The proposed mechanism behind this kinetic observation involved increased affinity of GABA for the receptor [46]. Subsequent whole cell data demonstrated an isolated left-shift in the GABA concentration-response curve without affecting the macroscopic current shape (within the limitations of slow oocyte perfusion) [15]. Although binding studies have also consistently shown that BDZs increase the affinity for GABA, the measurement of binding affinity can be contaminated by effects on gating [8]. 

One implication of an affinity-based mechanism is that the observed functional effects of BDZ modulation could depend upon the GABA concentration. Modulating affinity is predicted, for example, to have markedly different impact on peak current amplitude measurements, depending on the agonist concentration being tested experimentally. This is most easily appreciated when considering a modulator causing a left-shift in the sigmoidal GABA concentration response curve: the enhanced current amplitude would be greater when tested at EC_30_ than EC_90_ GABA concentrations. The experimental conditions of the single channel studies involved prolonged exposure to low GABA concentrations that resembled those occurring in extrasynaptic or tonic signaling *in vivo* (persistent ~1 µM GABA exposure) [13, 16, 26, 49, 55]. Synaptic exposure to GABA, in contrast, differs in the duration (transient) and concentration (high) of GABA encountered during these phasic events [21]. How would GABA_A_ receptor single channel kinetics change in response to increased affinity under synaptic versus extrasynaptic conditions? 

## PREDICTED IMPACT OF CHANGING GABA AFFINITY UPON GABA_A_ RECEPTOR MACROSCOPIC CURRENT PROPERTIES.

The microscopic affinity of an agonist refers to the ratio of the binding to the unbinding rate constants, whereas affinity attributed to the EC_50_ of the concentration response curve or of binding studies is influenced by gating as well [8]. Whether modulating affinity involves k_on_ or k_off_, a strictly affinity-based mechanism of modulation predicts that BDZs will increase peak current amplitude only if receptors are activated by sub-saturating GABA concentrations. Once exposed to saturating agonist concentrations, the current amplitude ceiling would not be further increased by increasing GABA affinity. For similar reasons, macroscopic current desensitization observed during continued exposure to saturating GABA concentrations should not be altered by increasing GABA affinity [4]. The ceiling effect in both cases simply reflects the asymptotic approach to a fully liganded state of the receptors.

Experimental and simulation work has demonstrated that the current relaxation after GABA washout (deactivation) depends on multiple factors. Unlike peak amplitude and macroscopic desensitization, deactivation is sensitive to changes in affinity even when receptors are stimulated with saturating GABA. For brief saturating GABA exposure with instant washout (square pulse), deactivation should be sensitive to changes in GABA affinity only if they involve changes in the unbinding rate constant. Modulating the agonist binding rate constant could contribute to deactivation at synapses under conditions of sub-saturating GABA concentration or slow clearance of free GABA after synaptic release. However, if one assumes that there is negligible opportunity for rebinding of free GABA after the transient pulse, the predicted roles of k_on_ and k_off_ are straightforward and will be explored below. In concentration jump experiments using small cells lifted from the recording dish, rapid washout of free GABA is a reasonable assumption [1] (see below). 

**Fig. (1):**
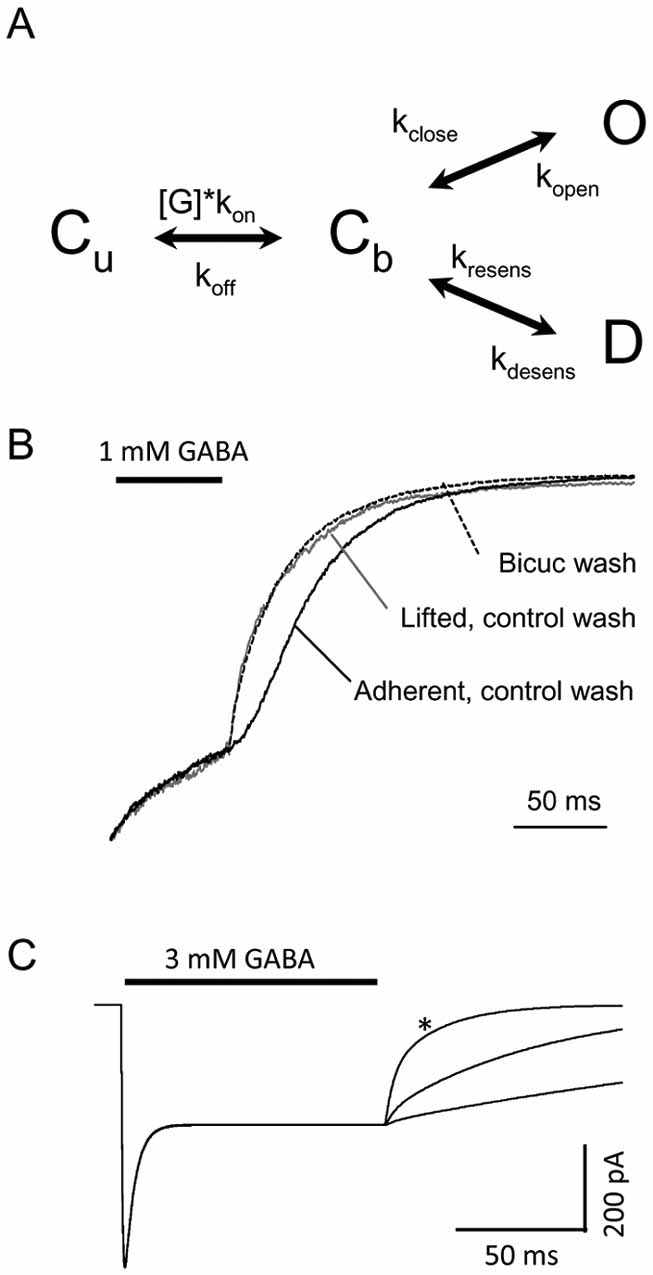
GABA_A_ receptor model and macroscopic currents. (**A**). 4- state kinetic model containing a single resting unbound closed state (C_u_), a bound closed state (C_b_),and one open (O) and one desensitized (D) state. Rate constants have units of s-1, except kon, which is in units of M^-1^s^-1^, as follows: k_on_ = 5 x 10_6_; k_off_ = 1000; k_open_ = 800; k_close_ = 500; k_desens_ = 800; k_resens_ = 100. (**B**). Experimental traces from α1β3γ2L receptors in a single whole cell patch clamp experiment. Only the terminal portion of each trace is shown (a 400 ms concentration jump in 1 mM GABA preceded the washout). *Solid line*, deactivation while cell remains adherent to the recording dish; note the slow time course reflecting GABA rebinding due to imprecise washout. *Dashed line*, deactivation while cell remains adherent, but had been jumped from GABA into bicuculline (1 µM), instead of control wash; note the sharper deactivation. *Gray line*, deactivation after cell was lifted from the recording dish, improving solution exchange (control wash); note the similarity with the bicuculline wash, as lifting the cell or exposing the intact cell to bicuculline similarly prevented GABA rebinding. (**C**). Increasing GABA affinity by decreasing k_off_ prolongs deactivation without altering the current shape during the GABA pulse (peak amplitude, desensitization). The asterisk indicates baseline parameters; slower deactivation occurred when k_off_ was decreased to 200 and 50 s^-1^. Adapted from reference [5] with permission.

Simplified models have proven useful for investigating the relationship between microscopic and macroscopic current kinetics [2, 4, 38]. Only 4 states are considered in this model: a resting closed state (closed-unbound, C_u_), a GABA-bound closed state (closed-bound, C_b_) accessed by a single GABA binding step, a GABA-bound open state (open, O), and an additional GABA-bound closed state (desensitized, D) (Fig. **1A**). The D state permits macroscopic desensitization to occur, depending on the GABA concentration and the relative rate constants associated with O and D [4, 38]. When simulated currents were evoked by saturating GABA concentrations, neither the peak amplitude, nor the macroscopic desensitization time-course was affected by increased affinity, whether achieved by increasing k_on_ (not shown) or by decreasing k_off_ (Fig. **1C**) [5]. The range of k_off_ variation discussed in these simulations were chosen to reflect the typical ~50% or greater slowing of the decay kinetics of deactivation [39, 5, 44], and are not meant to represent true GABA unbinding rate constants in recombinant or native receptors (Haas *et al.* [17] modeled k_off_ as 170/s). This result is expected since the gating portion of the model, which dictates the amplitude and desensitization time course, is insensitive to the binding step provided that saturating GABA is present. Decreasing k_off_ did prolong deactivation, as expected, whereas increasing k_on_ had no effect [5]. Thus, affinity-based prolongation of deactivation is restricted to the unbinding rate constant k_off_. This pattern holds for more comprehensive models provided that unbinding remains a terminal event [17, 27].

Single channel simulations offered an opportunity to determine how changes in affinity are predicted to impact opening frequency under synaptic and extrasynaptic conditions [5]. For the purposes of this analysis, opening frequency is defined operationally as the number of observed openings per unit time. Although some authorities refer to the inverse of brief closed duration(s) as the opening frequency, an alternative interpretation of brief (intraburst) closures is that they represent “distal” closed states. Work from the Macdonald laboratory has modeled 3 distinct opening rate constants per open state: one “standard” opening rate constant and two opening rate constants from the distal closed states [17]. Here, because the generic model used in the simulations has only one open state, and one rate constant leading to it, the distinction is not relevant. However, as we will see below, even in such a simple model, the opening rate constant is not equivalent to the opening frequency.

The simple kinetic model in Fig. (**1A**) is representative of a more general class of models with two important features: 1) unliganded receptors can only access “gating states” after ligand binds, and 2) once bound, receptors cannot unbind GABA while they are visiting gating states (in this case, states O and D). The first feature does not allow for spontaneous (unliganded) openings. Although such events clearly occur in certain circumstances, such as mutations or when an epsilon subunit is present [40, 48], they are low probability events in α1β3γ2L isoforms. The second feature also implies that unliganded gating does not occur, but refers specifically to gating in receptors that were already activated by and subsequently released GABA. In other words, in this class of models, because a receptor cannot continue gating after GABA has unbound, unbinding is considered the terminal event in the deactivation process. This latter feature has important implications for the simulations that follow. 

Although models containing “cyclic” features (in which agonist can bind to and unbind from any state) have been proposed, prior studies suggest that, at least for the α1β3γ2L isoform, all open and pre-open states must be GABA bound [1]. This conclusion was based on experimental demonstration that deactivation currents following GABA exposure were resistant to bicuculline block. Considerable opportunity for GABA rebinding exists during GABA washout, if cells are recorded while adherent to the culture dish, even in concentration jump experiments. This is shown in Fig. (**1B**), which compares the deactivation currents in three conditions: adherent cell, control wash; adherent cell, bicuculline wash, and lifted cell, control wash. The inverse agonist bicuculline antagonizes open channels only when GABA is not bound (such as mutation-induced spontaneous openings, or direct activation with other agents such as neurosteroids). When rebinding opportunity is minimized by lifting the cell, bicuculline antagonism of deactivation currents is also minimized. This finding also indicates that the contribution of unliganded channels (those that have unbound GABA) to the openings that constitute deactivation currents can be taken as negligible, and unbinding can be considered as a terminal event in the kinetic scheme as described above. 

A complementary experimental argument against k_on_ modulation underlying a BDZ-induced change in GABA affinity involved similar double jump experiments as described above with bicuculline. Demonstrating enhancement of current deactivation by a modulator applied selectively during GABA_A_ receptor deactivation – that is, with GABA bound and channels visiting gating states, but in the absence of free GABA, precludes an effect on k_on_ [1]. In those experiments, diazepam delivered selectively during the deactivation process enhanced the current, consistent with an effect on k_off_ but incompatible with an effect on k_on_. 

## SINGLE CHANNEL SIMULATIONS UNDER EXTRASYNAPTIC CONDITIONS.

**Fig. (2):**
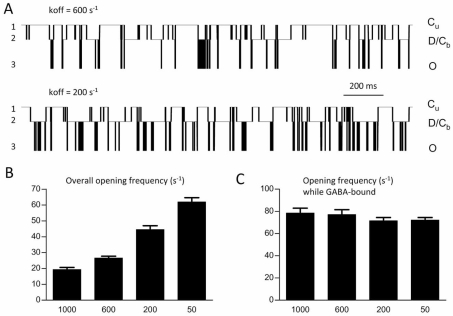
**Simulated single channels under extrasynaptic conditions. (A)**. Single channels were simulated such that unbound closed (C_u_), bound closed (C_b_ and D), and bound open (O) states are distinguished via different conductance levels (levels 1, 2 and 3, respectively) (QUB software,  www.qub.buffalo.edu). Notice the increased time spent in bound states when k_off_ is decreased (lower panel). Repeated 2 second exposures to 1 μM GABA were used to generate the data summarized in panels B and C. The Markov states corresponding to the conductance levels (1-3) are also shown on the right of traces in panel A and B. (**B**). Overall opening frequency increased as affinity was increased (*via* decreasing k_off_). (**C**). When frequency analysis is restricted to times when GABA is bound, it is unchanged by decreasing k_off_. Adapted from reference [5] with permission.

The majority of extrasynaptic GABA_A_ receptors contain the δ subunit, which renders them insensitive to BDZs [33, 34]. However, receptors containing a γ subunit are sometimes targeted to non-synaptic membranes in regions such as the hippocampus [16]. Thus it is important to consider how BDZ modulation of GABA_A_ receptor channels might manifest under extrasynaptic conditions, which may serve important physiological roles [26, 42, 49]. Single channel simulations using the simple model above allowed unambiguous determination of the occupation of different types of closed states by assigning artificial conductance levels (Fig. **2A**). In actual single channel experiments, multiple kinetic states with the same conductance (and therefore indistinguishable by amplitude criteria) can be resolved statistically through analysis of the duration histogram of many events. However, the question we wanted to answer required kinetic information available only *in silico*: the exact moment of GABA unbinding. Here, the imposed conductance levels allowed distinction between occupancy of the unbound state (C_u_, level 1) versus the GABA-bound non-conducting states (C_b_ and D, level 2) and conducting state (O, level 3). This strategy overcomes a critical limitation for testing the above hypothesis for an affinity-based BDZ mechanism: one cannot experimentally measure the exact time of agonist dissociation from a single channel, which is required to calculate the opening frequency under synaptic conditions. In other words, one would not know when to “stop looking” after delivering a brief GABA pulse, in order to calculate openings-per-time. 

By decreasing k_off_, BDZs would decrease the likelihood of a channel entering the unbound state C_u_, which is long-lived at low GABA concentration. This would result in decreased average closed time, which would indirectly increase opening frequency. Simulations confirmed this prediction when frequency was measured across a range of affinity (k_off_) parameters (Fig. **2B**). However, if the analysis of openings is restricted to the time when the channel was GABA-bound (which cannot be done experimentally), opening frequency was not increased as affinity was changed (Fig. **2C**). This result confirms that the change in frequency seen in Fig. (**2B**) derives from the decrease in time spent in the unbound state. The small trend toward decreased frequency may relate to the fact that decreasing k_off_ increases the lifetime of C_b_, which contributes to the GABA-bound closed time, and therefore impacts calculations of opening frequency (however this change is small relative to the overall bound-closed time which is dominated by the D state). 

## SINGLE CHANNEL SIMULATIONS UNDER SYNAPTIC CONDITIONS

**Fig. (3):**
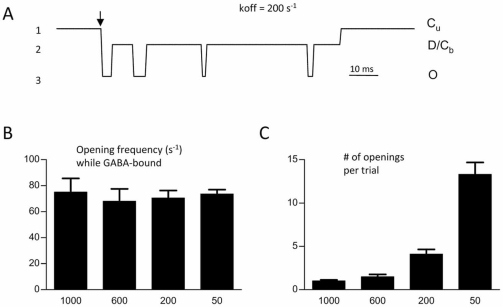
** Simulated single channels under synaptic conditions. (A)**. Simulated single channel response to a 1 ms GABA pulse (1 mM), using the same model as in Figure 2. The Markov states corresponding to the conductance levels (1-3) are also shown on the right of the trace. (**B**). The number of openings recorded after the brief synaptic GABA pulse increased as affinity increased (by decreasing k_off_). (**C**). The frequency of openings was, however, unchanged across the same range of k_off_. Adapted from reference [5] with permission.

Consider the extreme case of synaptic GABA release that rapidly reaches peak concentration and then rapidly falls to zero concentration (a square pulse). Even if receptors are not saturated, if GABA is immediately cleared from the synaptic cleft, no rebinding can occur and the post-synaptic current time-course will reflect transitions among GABA-bound states. In this case, the context resembles the above analysis in which frequency calculations were restricted to times when GABA was bound – which predicts no change in frequency under synaptic conditions. Simulated brief (1 ms) square pulses of high GABA concentration, mimicking phasic synaptic events, confirmed this prediction (Fig. **3**) [5]. The opening frequency was not increased by simulating BDZ modulation acting to slow the unbinding rate constant (Fig. **3B**). However, as expected, slower unbinding increased the total number of openings (Fig. **3C**), simply by virtue of additional time to access bound states before the terminal unbinding event occurred. 

Although the precise time-course of GABA clearance may be variable under synaptic conditions, the slowed GABA unbinding that increases the number of openings may be the dominant mechanism of BDZ-mediated prolongation of synaptic currents. Exceptions to this type of “saturating square pulse” synaptic simulation certainly exist, either because peak concentrations are not saturating, or because of slow re-uptake or spillover that allow sub-saturating concentrations to linger in the cleft [44, 45]. Although the conclusions are unchanged by non-saturating pulses, under circumstances of lingering free GABA, a mixture of increased frequency and increased number of openings can be expected, in proportion to the relative opportunities for rebinding before GABA is finally cleared. Also, the precise concentration of neurotransmitter remaining in the synaptic cleft between vesicular release events is uncertain. This potential residual cleft concentration may be regulated by different factors than those controlling extrasynaptic concentrations, as well as the equilibrium between the cleft compartment and neighboring extrasynaptic space. The precise concentration profile of GABA at central synapses remains uncertain [16, 18, 21, 36, 41, 43, 44]. Nevertheless, the central conclusion is that the manifestation of changing affinity upon measurement of opening frequency is critically dependent upon the extent to which unbinding and rebinding of GABA can occur. When rebinding can occur, the opening frequency increases as affinity increases, but when it cannot occur, the increase in affinity leads to an increased number of openings without increasing their frequency. 

## GABA_A_ RECEPTOR DEACTIVATION DEPENDS ON MULTIPLE FACTORS.

The simulations demonstrate that BDZs extend the opportunity for individual receptors to transition among GABA-bound states, leading to prolonged deactivation after brief (synaptic) pulses by increasing the number of openings. Unlike desensitized states, which can also prolong deactivation by increasing the average time receptors are GABA-bound [4, 20], decreasing k_off_ will necessarily increase charge transfer. Increasing stability of desensitized states will accelerate early deactivation, and prolong the slow portions of deactivation. 

**Fig. (4):**
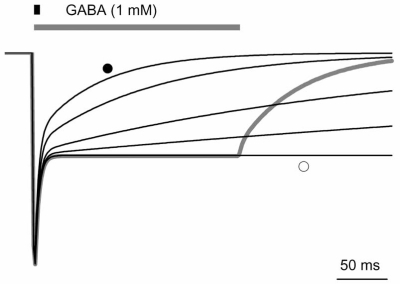
Macroscopic desensitization sets the limit of k_off_-mediated slowing of deactivation. Simulated macroscopic currents (solid lines) evoked by 1 ms pulses of 1 mM GABA, across a range of k_off_ values (200, 100, 30, 10, and 0). k_off_ = 200 is indicated by the solid circle, while k_off_ = 0 is indicated by the open circle. A typical BZDinduced prolongation is represented by the koff value of 100 (adjacent to koff = 200, indicated by the solid circle). These are shown in comparison with a 200 ms pulse of 1 mM GABA (gray line). Notice that the progressive slowing of deactivation following the brief pulses approaches a limit set by the macroscopic shape of the current during prolonged exposure.

For non-cyclic models such as Fig. (**1A**), deactivation is predicted to be sensitive to changes in every rate constant except k_on_ (except theoretically when changes in k_on_ render a given GABA concentration likely to produce predominantly mono-liganded receptors, which may have access to distinct gating states in 2-binding site models). Although changes in open and desensitized states fundamentally change the shape of deactivation, the effects of modulating k_off_ appear to provide a mechanism to slow the rate of deactivation up to a boundary defined by the macroscopic desensitization time course [4]. The macroscopic desensitization time-course with prolonged saturating GABA exposure defines this limit such that, for a simulated synaptic pulse when k_off_ = zero (infinite affinity), the current does not exceed the macroscopic desensitization time-course (Fig. **4**). Increasing the stability of open states will also prolong deactivation, with concomitant effects on macroscopic desensitization (and therefore the deactivation boundary for affinity related changes) [2, 4]. Likewise, changing the stability of desensitized states alters deactivation, as well as macroscopic desensitization. However, for any given set of open and desensitized stability parameters, the enhancement of deactivation from changes in k_off_ will be limited by the macroscopic time course of desensitization. 

## SINGLE-CHANNEL OPENING FREQUENCY DEPENDS ON MULTIPLE FACTORS

Although the term “mechanism” is sometimes used when describing changes in single-channel opening frequency due to a mutation or allosteric modulator, opening frequency is more accurately described as an observation because it is influenced by several microscopic mechanisms. Observing changes in opening frequency does not uniquely map to a specific kinetic mechanism, and the direction of change in frequency does not necessarily even correlate with efficacy in terms of total charge passed per time (that is, frequency and efficacy can be “uncoupled”). For example, consider a “flickering” open channel blocker, which could dramatically increase opening frequency despite being an antagonist that decreases the charge passed per time. Similarly, an agonist that markedly increased open duration may indirectly decrease opening frequency, despite increasing the charge passed per time. Considering the above 4-state model, reducing the average occupancy of the D state (either by decreasing the entry rate or increasing the exit rate) will increase opening frequency, as well as gating efficacy (although deactivation will be faster). Increasing the D state occupancy will have the opposite impact. For increases in the open duration (by decreasing k_close_), the limit of opening frequency cannot be faster than the inverse of the mean open duration. One can imagine a case of prolonging open durations such that opening frequency decreases, yet efficacy in terms of charge passed per time increases. Note that the opening rate (k_open_) is therefore not the only factor determining the observed opening frequency. In the case of BDZs, a single mechanism, increased affinity *via *decreasing k_off_, is predicted to have different functional impacts on opening frequency, depending on the context in which receptors are activated. 

## EVIDENCE THAT BDZ MODULATION INVOLVES MORE THAN GABA AFFINITY

Studies investigating changes in reaction rates of engineered cysteine residues at or near those implicated in GABA binding demonstrated movement in response to BDZs [23], consistent with an affinity-based mechanism of enhancement. However, certain experimental findings suggest that BDZ modulation may also affect GABA_A_ receptor gating. Chimeric γ-δ subunit analysis suggested that regions distant from the putative GABA binding pocket, near the first two transmembrane domains, as well as the TM2-TM3 intracellular loop, were important for BDZ modulation [19]. Cysteine modification experiments also implicated movement in TM3 residues in response to BDZ (which did not appreciably activate the receptors in that study, suggesting these movements were not related exclusively to spontaneous gating) [56]. These reports suggest that BDZ modulation involves structural alterations that could influence channel gating. 

In addition to these structural investigations, functional studies from several groups suggest BDZ modulation may influence channel gating. For example, Mercik *et al.* showed (in addition to a likely effect on GABA affinity), small effects of zolpidem (but not flurazepam) on desensitization, and both zolpidem and flurazepam resulted in smaller maximal GABA-evoked currents [35]. These effects have not been seen with BDZs in other studies however [5, 28]. BDZs have been shown to enhance spontaneous GABA_A_ receptor currents from mutated receptors by several groups [5, 6, 11, 51]. In some cases, possibly requiring high receptor expression levels, spontaneous currents from wild type α1β3γ2L receptors (which are typically very small compared to GABA evoked currents) can be recorded and are enhanced by BDZs [1]. New generation BDZ binding site modulators also enhance spontaneous currents, as zolpidem increased the spontaneous activity generated by the α-subunit pore mutation L263S [11]. Further support for effects on channel gating derived from the finding that diazepam increased the efficacy of GABA_A_ receptor partial agonists; enhanced efficacy of partial agonists using the BDZ modulator chlordiazepoxide has been previously reported as well [32]. Rusch and Forman also demonstrated BDZ modulation of spontaneous currents resulting from α1(L264T) mutation, and showed that efficacy of a partial GABA_A_ receptor agonist was increased by the BDZ midazolam [48]. 

Simplified allosteric models (usually with one open state, one closed state, and no desensitization) have been proposed to account for these BDZ modulation results, involving a shift toward stabilizing the open/active conformation. However, there are several important features of the proposed Monod-Wyman-Changeux type models that are worth considering. First, they typically do not account for multiple open states, multiple closed states, or macroscopic desensitization. Using simplified models with these limitations to generate concentration response curves without taking into account these features of GABA_A_ receptor kinetics [48] is of uncertain utility, given the potential impact of these states (in particular, the desensitized states). Although desensitized (and additional open) states could be included in these types of models, the connectivity and stability of D states can have non-intuitive effects on macroscopic current behavior even when cycles are not present [4]. Second, the multiple embedded cycles that characterize these models makes maintenance of detailed balance difficult, and failure to constrain microscopic reversibility [22] implies an energy-requiring mechanism of uncertain source. Finally, this class of model involves binding cycles that explicitly allow GABA to bind and unbind from all states, including open states. In fact, the agonist efficacy in such models is directly related to the affinity differential between the open and resting states. However, such cycles are difficult to reconcile with data suggesting that GABA does not unbind from (or, by inference, bind to) open or pre-open states [1], as described above. Although this finding is consistent with any non-cyclic model of the class exemplified by Fig. (**1A**), the cyclic models predict the opposite finding, which is that bicuculline should inhibit the deactivation current in proportion to the availability unliganded open (or pre-open) receptors. If such receptor conformations are noncontributory (unstable once GABA unbinds), then the MWC model appears to collapse to a non-cyclic model. More complex linear models have been proposed for αβγ and αβδ isoforms, which generate realistic current behavior under single channel, macroscopic, concentration-response curve, and allosteric modulator conditions [14, 17, 27]. 

The finding of enhanced spontaneous currents in wild type and mutated channels appears difficult to reconcile with a BDZ mechanism that is strictly limited to GABA binding affinity, as pointed out by Rusch and Forman [48]. One potential explanation for this apparent disparity is that spontaneous gating involves distinct conformations compared to liganded receptor gating. For example, consider a spontaneous transition from the state C_u_ in Fig. (**1A**) to an isolated open state, which can be accessed with low probability at baseline, and which can be favored by mutation or by the action of allosteric modulators. BDZs could influence this transition, but in the presence of GABA the affinity mechanism would dominate. Although it would not be surprising for careful study of any allosteric modulator to reveal effects on multiple kinetic processes, the simulations discussed above nevertheless point to distinct predictions of altered affinity (*via *k_off_) upon GABA_A_ receptor kinetics under synaptic versus extrasynaptic conditions. 

## ACKNOWLEDGEMENT

This review was not funded. The author acknowledges Dr Robert L. Macdonald, in whose lab the reviewed experiments were undertaken.
